# The Role of Extracellular Matrix Remodeling in Skin Tumor Progression and Therapeutic Resistance

**DOI:** 10.3389/fmolb.2022.864302

**Published:** 2022-04-26

**Authors:** Julia E. Fromme, Paola Zigrino

**Affiliations:** ^1^ Department of Dermatology and Venereology, University of Cologne, Faculty of Medicine and University Hospital Cologne, Cologne, Germany; ^2^ Mildred Scheel School of Oncology Aachen Bonn Cologne Düsseldorf (MSSO ABCD), Cologne, Germany

**Keywords:** skin, melanoma, SCC, MCC, BCC, therapy, ECM, TME

## Abstract

The extracellular matrix remodeling in the skin results from a delicate balance of synthesis and degradation of matrix components, ensuring tissue homeostasis. These processes are altered during tumor invasion and growth, generating a microenvironment that supports growth, invasion, and metastasis. Apart from the cellular component, the tumor microenvironment is rich in extracellular matrix components and bound factors that provide structure and signals to the tumor and stromal cells. The continuous remodeling in the tissue compartment sustains the developing tumor during the various phases providing matrices and proteolytic enzymes. These are produced by cancer cells and stromal fibroblasts. In addition to fostering tumor growth, the expression of specific extracellular matrix proteins and proteinases supports tumor invasion after the initial therapeutic response. Lately, the expression and structural modification of matrices were also associated with therapeutic resistance. This review will focus on the significant alterations in the extracellular matrix components and the function of metalloproteinases that influence skin cancer progression and support the acquisition of therapeutic resistance.

## Introduction

The extracellular matrix (ECM) represents the non-cellular compartment of the microenvironment in all tissues and organs that serves as a scaffold, rendering structure and stability (Clause and Barker, 2013). In healthy human skin, the dermal ECM contains several molecules, including collagens, glycoproteins (such as fibronectin (FN) and tenascins), proteoglycans (such as versican and decorin), and glycosaminoglycans (such as dermatan sulfate and hyaluronan), all forming a complex network that provides structural integrity (Pfisterer et al., 2021). The ECM network is continuously remodeled and modified by non-enzymatic (e.g., glycation and carbonylation) and enzymatic reactions (Frantz et al., 2010). These contribute to the ECM mechanical properties, including elasticity, tensile strength, and tissue stiffness, regulating cellular functions, including cellular proliferation, apoptosis, and differentiation (Frantz et al., 2010; Clause and Barker, 2013). The homeostasis of the skin ECM is maintained through the tightly regulated spatiotemporal coordination of production, deposition, and turnover. Corruption of this highly dynamic process leads to altered balance that underlies the pathogenesis and progression of many diseases (Winkler et al., 2020). Matrix metalloproteinases (MMPs) have critical roles in this process, and imbalanced activity is implicated in the pathogenesis and progression of many skin diseases, e.g., inflammatory diseases, healing defects, fibrosis, and cancer (Zigrino and Mauch, 2017).

In cancer, ECM component synthesis, modification, and degradation dynamics are deregulated, leading to altered composition, density, and mechanical properties (Lu et al., 2012). In various solid tumors, fibroblasts are activated by these alterations. In turn, the generated CAFs produce more matrix proteins and build a desmoplastic reaction, thereby altering the biophysical properties of the peritumoral stroma. A prominent desmoplastic response can be seen in pancreatic cancer and some skin cancers (Neesse et al., 2015; Tschumperlin and Lagares, 2020). A denser peritumoral matrix may reduce tumor vascularization and enhance interstitial pressure, thus preventing drug permeation into tumors (Stylianopoulos, 2017). Furthermore, as seen in HNSCC, mechanical stimuli from the ECM can modulate tumor metabolism and the interaction of cancer and stromal cells within the tumor niche to promote growth and aggressiveness (Bertero et al., 2019).

The tumor microenvironment (TME), encompassing the complex network surrounding the tumor, consists of non-cellular (ECM) and cellular components. Many cells of the tumor microenvironment contribute to the production and deposition of ECM proteins; however, the primary producer of the ECM is fibroblasts (Hinz et al., 2012). Once remodeled, the tumor microenvironment activates signaling pathways that stimulate tumor growth, migration, and invasion. Solid tumors are often desmoplastic (dense fibrosis around the tumor) due to altered organization, reduced turnover, and enhanced post-translational modifications of ECM components (Frantz et al., 2010; Lu et al., 2012). In these processes, a variety of proteolytic enzymes are involved, and among the best-studied are the matrix metalloproteinases (MMPs) and a disintegrin and metalloproteinases (ADAMS) (Egeblad and Werb, 2002). These enzymes are pivotal in promoting tumor cell invasion and metastasis in various ways. They can remodel and degrade ECM components, increase the availability of growth factors and release bioactive ECM peptides, the so-called matrikines (Sternlicht and Werb, 2001; Moro et al., 2014). The proteolysis of ECM components have both tumor-promoting and -antagonizing effects (López-Otín et al., 2009; Ford and Rajagopalan, 2018). For example, matrikines, such as endostatin a proteolytic product from type XVIII collagen, or those derived from elastin or plasminogen (angiostatin) cleavage, are anti-angiogenic and can act in a tumor-promoting or suppressing manner (O'Reilly et al., 1997; Sim et al., 1997; Da Silva et al., 2018). During tumor cell migration into the tissues, the majority of the ECM-degrading events are supportive as they generate paths and leave space for the deposition of the new tumor-derived ECM components that facilitates progression (Wolf and Friedl, 2009). Changes in the composition and properties of the tumor ECM contribute to modifying the fundamental cellular processes of tumor cells and resident or chemoattracted inflammatory cells (Winkler et al., 2020).

**FIGURE 1:**
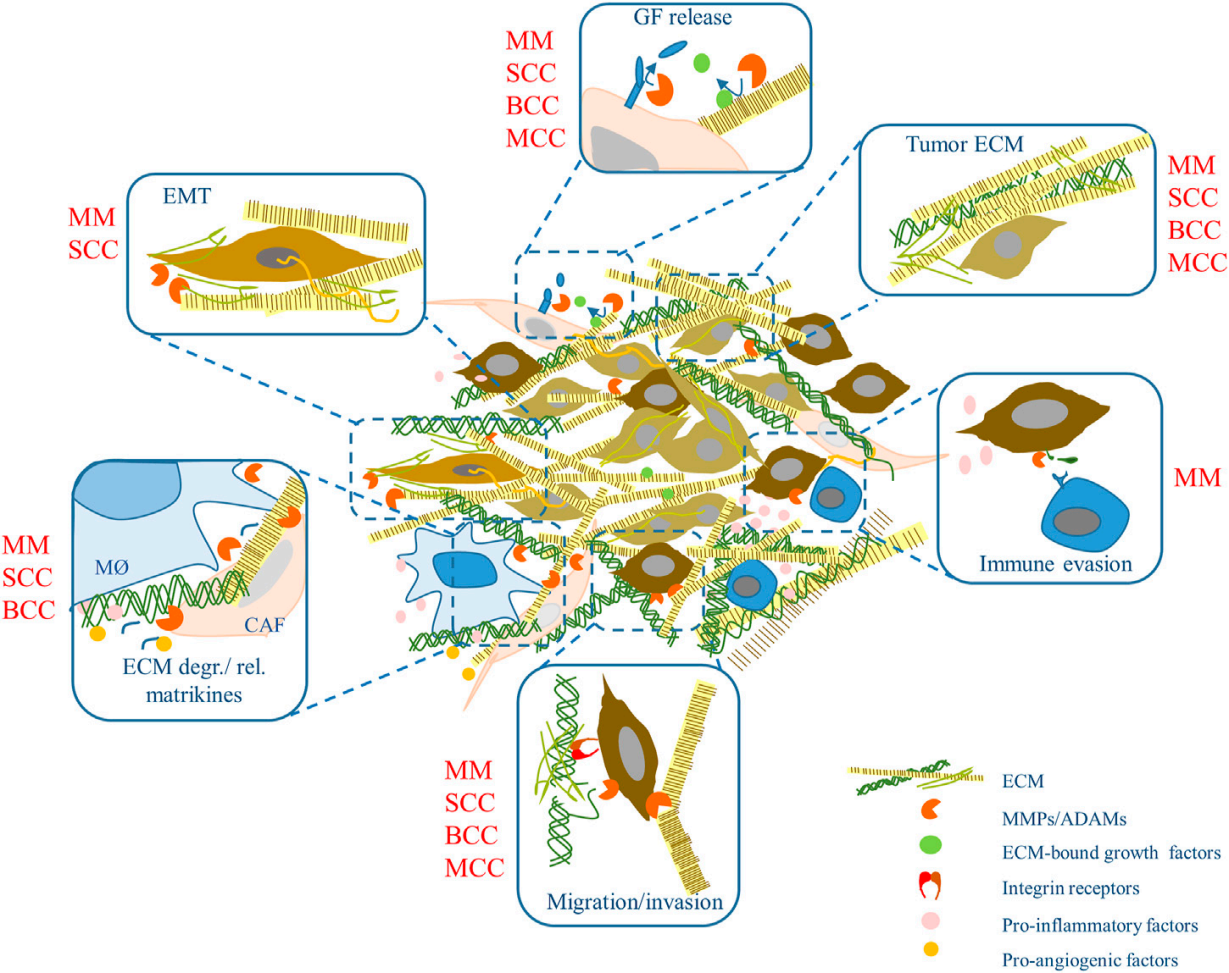
ECM remodeling (and more) in skin cancer. ECM components and metalloproteinase activities may influence skin tumors development and progression modulating a variety of events. Some of these were shown in specific skin tumors (in red). MM, malignant melanoma; SCC, squamous cell carcinoma; BCC, basal cell carcinoma; MCC, Merkel cell carcinoma. MØ, macrophages; GF, growth factor; CAF, cancer-associated fibroblasts; EMT, epithelial-to-mesenchymal transition.

The cellular part of the tumor microenvironment includes a variety of cells: cancer-associated fibroblasts (CAF), epithelial, endothelial, and immune cells such as macrophages, dendritic cells, myeloid-derived suppressors, and T cells (CD4^+^, CD8^+^, and Tregs) (Ji et al., 2020). These cells interact in continuous crosstalk displaying enormous phenotypic plasticity (Avagliano et al., 2020) and supporting tumor growth and invasion. A general overview of the role of different ECM remodeling mechanisms in the various skin cancers is summarized in Figure 1.

In the following paragraphs, we will summarize the available data on the ECM and metalloproteinases playing a role in the progression of the most frequent skin cancers such as melanoma, squamous cell carcinoma (SCC), Merkel cell carcinoma (MCC), and basal cell carcinoma (BCC). In addition, we will highlight the emerging roles of ECM proteolytic remodeling for the acquisition of drug resistance in the various skin tumors undergoing two to date successful treatments, MAPK and checkpoint inhibitors.

## ECM and Remodeling Enzymes in the Progression of Skin Cancers

### Melanoma

Melanoma is one of the deadliest cancers, with about 7,000 estimated deaths in the United States in 2021 representing 80% of all skin cancer deaths (Romano et al., 2021; National-Cancer-Institute, 2022). Unlike breast, lung, and colorectal cancer, where desmoplasia (the growth of connective tissue) is strongly linked to the tumor’s aggressiveness (Chan et al., 2019), melanoma does not display a prominent desmoplastic response around tumors. Only a specific subtype, desmoplastic melanoma, has a prominent fibroplasia but a better prognosis (Busam, 2011). However, melanoma growth and metastasis strongly depend on the ECM composition. Indeed, in skin aging, age-alterations in the ECM, including decreased collagen density and fragmentation, promote melanoma migration and metastasis but hamper infiltration of CD4^+^ and CD8^+^ T cells (Ecker et al., 2019; Kaur et al., 2019). The alterations underlying these effects include, in addition to the decreased collagen density, increased alignment of fibers, and the reduced fibroblasts’ expression of the hyaluronan and proteoglycan link protein 1 (HAPLN1), a cross-linking protein that stabilizes proteoglycan monomer aggregates with hyaluronic acid (Kaur et al., 2019). Reduced HAPLN1 in aged skin destabilizes the ECM and perturbs VE-cadherin connections between the lymphatic endothelial cells, thus increasing permeability and promoting melanoma metastasis and extravasation from lymphatic vessels (Ecker et al., 2019). However, the role of collagen in melanoma growth is not completely clear. Enhanced expression of collagen I in the stroma of melanoma is associated with higher invasiveness and is a predictor of worse prognosis (van Kempen et al., 2008; Miskolczi et al., 2018). In line with these, it was recently seen that primary melanomas arising over UV-induced collagen degradation in the skin are less invasive and reduced invasion improves survival (Budden et al., 2021).

In contrast, other studies show that increased tissue stiffness and fibrillar collagen abundance can negatively affect melanoma cell growth and migration (Liu et al., 2012b; Li Y. et al., 2019). Furthermore, in 50% early and 69% advanced-stage melanoma, the *COL1A2* promoter is methylated, resulting in reduced expression of transcripts (Koga et al., 2009). The controversial role of collagen and stiffness in melanoma is possibly due to factors that have not always been considered when analyzing tissues and isolated cells, such as tumor stage, type, age, gender, skin location, mutation status, and exposure to external insults. More detailed investigations are required.

Among the ECM components enhanced in melanoma is tenascin C (TNC), which mediates melanoma growth and metastatic spread (Shao et al., 2015). It can bind to various integrin receptors, and its cleavage by matrix metalloproteases and serine proteases can generate cryptic sites exposing new adhesive sequences for cell adhesion receptors (Midwood and Orend, 2009). Fibronectin (FN) is also crucial for the metastatic spread of melanoma cells, it is regulated by ERK/MAPK signaling, modulates the process of epithelial-to-mesenchymal transition, regulates apoptosis, and supports cell invasion (Li B. et al., 2019); (Gaggioli et al., 2007). The microenvironment of human melanoma expresses a variety of additional matrix proteins, including collagen XIV (Pach et al., 2021), hyaluronan (Gebhardt et al., 2009), laminin 332 (Pyke et al., 1994), and several others identified in the matrisome (matrisome is defined as a combination of ECM proteins and associated factors) of human melanoma xenografts (Naba et al., 2012).

In addition to the production of ECM components, tumor and stromal cells express and activate several proteolytic enzymes (Labrousse et al., 2004). This allows cells to proteolytically process different ECM components, rearrange them, release bound growth factors and cellular bonds to invade the underlying tissue. Several matrix metalloproteinases are expressed in melanoma, the tumor, and the peritumoral stroma, including MMP-1, MMP-2, MMP-9, MMP-13, and MMP-14 (Zigrino and Mauch, 2017). Shaverdashvili et al. (2014) demonstrate *in vivo* that melanoma cells expressing MMP-14 activate MMP-2, thereby maintaining RAC1 activity and promoting the invasive capacity of melanoma cells. These data explain melanoma cells’ inability to form distant metastasis *in vivo* when MMP-14 expression is suppressed. In other tumor cells, fibrosarcoma, inhibition of MMP-14 may indirectly, by reducing FN lysis, lead to formation of stable focal adhesions, stronger cell adhesion to collagen type I, FN, and laminin, and reduced cell migration (Takino et al., 2006). The interaction between MMP-14, MMP-2, and laminin 332 γ two chains in highly invasive melanoma also promotes vascular mimicry, inducing melanoma progression (Seftor et al., 2001). MMP-14 and MMP-2 are active in melanoma cells and the neighboring stroma, and their co-localization correlates with melanoma progression (Hofmann et al., 2000). High MMP-14 expression and active MMP-2 are predominantly found in highly invasive cell lines, leading to FN processing and promoting invasion (Kurschat et al., 1999; Sato and Takino, 2010; Jiao et al., 2012). Given the role of MMP-14 in melanoma progression, Devy et al. blocking MMP-14 in an *in vivo* model of murine melanoma metastasis could reduce metastasis formation (Devy et al., 2009). In melanoma patients, high MMP-14 expression has been linked with poor prognosis (Wu et al., 2014), and MMP2 expression is a negative prognosticator independent of tumor thickness and ulceration (Rotte et al., 2012). Also, in patients with mucosal melanoma, MMP-14 represents a negative prognosticator. It is upregulated in BRAF and NRAS mutated melanoma, promoting the progression of these tumors (Iida et al., 2004; Kondratiev et al., 2008).

MMP-1, a collagenase, is produced by melanoma cells and peritumoral fibroblasts, increasing tumor growth and metastasis. Interestingly, when made by fibroblasts, it cleaves PAR1 (thrombin receptor), thereby inducing PAR1-dependent Ca2+ signals and enhancing the metastatic capacity of cancer cells (Wandel et al., 2000; Boire et al., 2005; Blackburn et al., 2009; Moro et al., 2014). Another collagenase, MMP-13, is increased during melanoma invasion, and its stromal expression is required for melanoma vascularization (Airola et al., 1999; Zigrino et al., 2009).

Expression of MMP-3 and MMP-8 was associated with a worse prognosis in melanoma (Nikkola et al., 2002; Vihinen et al., 2008). The collagenase MMP-8 is mainly found in patients with ulcerated and angio-invasive primary tumors (Vihinen et al., 2008).

Also, the expression of an ADAM protease, ADAM9, is associated with human melanoma progression (Alonso et al., 2007). This protease with adhesive domains is located at the invasive front of melanoma intra- and peritumoral (Zigrino et al., 2005). Further studies demonstrated that ADAM9 in melanoma cells is required to cleave the laminin beta 3 chain necessary to invade basement membranes and metastasis (Giebeler et al., 2017). Through its adhesive domains, ADAM9 on tumor cells interacts with integrin receptors at the surface of platelets to metastasize to the lung (Mammadova-Bach et al., 2016). In peritumoral areas, fibroblasts-melanoma interactions mediated by ADAM9 generate matrix modifications and enhance proteolytic activities necessary for melanoma growth (Zigrino et al., 2011; Abety et al., 2020).

### Cutaneous Squamous Cell Carcinoma

Cutaneous squamous cell carcinoma (cSCC) is the second most frequent skin cancer displaying a substantial metastatic potential. The peritumoral stroma of cSCC contains many fibroblasts, which become activated into CAFs that dominate the tumor microenvironment of SCC; these are the leading producers of ECM components (Öhlund et al., 2014). CAFs are characterized by the expression of, among others, fibroblast-specific protein 1 (FSP1, or S100A4), vimentin, and alpha-smooth muscle actin (alpha-SMA) (Somasundaram et al., 2016). They have a significant influence on the remodeling of the peritumoral matrix (Shiga et al., 2015). Structural changes in various matrix proteins such as collagens, laminins, and FNs were found in tumor samples of oral SCC (Ziober et al., 2006; Georgescu et al., 2020). Although data are available on the ECM role for growth and invasion of oral SCC and head-and-neck SCC, slightly less is known on cSCC. The matrisome’ profile of cSCC shows dysregulated ECM components and thereby altered cell-ECM interactions, associated with the increased metastatic potential of this cancer (Föll et al., 2018). Their analysis shows increased fibrinogen, collagen XVII, periostin (POSTN), and TNC in high-risk cSCCs resembling tissues with increased damage and repair. Low-risk tumors expressed more collagen I and cystatin A, the latter being necessary for epidermal differentiation (Brocklehurst and Philpott, 2013). Increased expression of collagen I is detected in transforming malignant keratinocytes and well-differentiated oral SCC (Stenbäck et al., 1999). By activating TGF-beta signaling, CAFs increase the expression of laminin 332 γ two chains in tumor cells, leading to tumor invasion (Siljamäki et al., 2020). Laminin 332 is enhanced in invasive head-and-neck SCC (Lyons and Jones, 2007), and expression of the γ two chains is increased at the invasive front of SCC (Coussens et al., 2000). Accordingly, the γ two chains, integrin beta 4 and collagen XVII overexpression, fostered migration and invasion of SCC cells in a tumor mouse model (Hamasaki et al., 2011). Loss of collagen XV and XVIII in actinic keratosis is an early sign of cSCC progression, and the remodeling of these proteins continues in progressing tumors (Karppinen et al., 2016). In cSCC, collagen IV is lower in the basement membrane than in BCC samples, in agreement with the higher metastatic potential of SCC (Kerkelä and Saarialho-Kere, 2003). In human cSCC, deposition of FN is enhanced in peritumoral areas of HPV (human papilloma virus) positive tumors, compared to negative cSCC (Heuser et al., 2016). This suggests that FN may result from a virus-induced reaction in the stroma.

Development of aggressive and metastatic cSCC is also detected in patients with recessive dystrophic epidermolysis bullosa (RDEB) (Fine et al., 2009). In these patients, the absence of collagen VII and the generation of a permissive stroma drive accelerated cSCC progression. SCC is developed in a tumor-primed dense and stiff matrix derived from the injured human RDEB skin rather than induced by the developing tumor (Mittapalli et al., 2016). Thus, it is a condition in patients causing mechanical tissue alterations and enhancing inflammation prone to cSCC development. For example, patients with various forms of fibrosis, displaying increased inflammation, have an increased risk for non-melanoma types of skin tumors (Hill et al., 2003).

Several MMPs, produced by stromal fibroblasts or transformed keratinocytes, are expressed in cSCC in tissue *in vivo* and cSCC cells *in vitro,* and these are extensively reviewed elsewhere (Riihilä et al., 2021). In addition to the significant contribution of CAFs, cells of the immune system also impact the proteolytic environment of cSCC. Pettersen et al. reported that in SCC CD163+ tumor-associated macrophages release MMP-9 and MMP-11 to enhance matrix turnover (Pettersen et al., 2011). Increased release of these proteases and MMP-10 by tumor-associated macrophages contributes to enhanced matrix turnover and angiogenesis, allowing for metastatic spread (Kambayashi et al., 2013; Georgescu et al., 2020). Naturally, besides releasing proteolytic enzymes, tumor-associated macrophages attract other immune cells generating a proinflammatory milieu that promotes SCC progression (Tampa et al., 2018).

### Basal Cell Carcinoma

Basal cell carcinoma (BCC) is the most frequent cancer occurring on the skin of elderly individuals, and the growth of BCC is mainly dependent on a supportive microenvironment (Van Scott and Reinertson, 1961). Using NGS technology, in basal cell carcinoma were identified 65 differentially expressed genes coding for ECM components and CAF markers such as fibroblast activation protein alpha (FAP-alpha) and platelet-derived growth factor receptor beta (PDGFR-beta) (Omland et al., 2017). Although BCC is rarely metastatic, matrix remodeling is crucial for local growth and invasion of these carcinoma cells and can be induced by crosstalk between tumor cells and CAFs in the peritumoral stroma (Micke et al., 2007; Omland et al., 2017). The enhanced matrix turnover induced by CAFs is driven by several matrix metalloproteinases (MMP-1, MMP-3, MMP-8, MMP-9) (Ciążyńska et al., 2018). MMP-1 is the critical collagen-processing proteolytic enzyme in BCC (Yucel et al., 2005). Its activity led to the accumulation of collagen fragments that were inefficiently cleared by gelatinases and feedback to fibroblasts to produce more MMPs to facilitate BCC growth (Yucel et al., 2005). MMP-2 and MMP-9 are increased in BCC in correspondence with areas where collagen type I and -IV degradation occur although these data are of transcript expression and thus conclusion is only correlative (Goździalska et al., 2016). Similarly, ADAM10, ADAM12, ADAM17 have been detected at the invasive fronts of BCC and displayed a different expression pattern in histologic subtypes of BCC, suggesting a role in BCC pathogenesis (Oh et al., 2009). In addition, the expression and function of a variety of other MMPs have been described and summarized elsewhere (Riihilä et al., 2021).

In addition to the production of ECM components and proteolytic enzymes, CAFs in the microenvironment of BCC increase the expression of tumor-relevant chemokines. Among those are chemokines promoting tumor progression and immunosuppression (CCL17, CXCL12) (Omland et al., 2017). In addition, tumor-associated macrophages play a crucial role in the BCC microenvironment. The proinflammatory M1 and anti-inflammatory M2 macrophages are balanced in nodular and fibrosing BCC (Kaiser et al., 2018). However, in highly invasive BCC, increased macrophage infiltration induces MMP-9 secretion leading to activation of the p38 MAPK/NF-kB/COX-2 axis (Tjiu et al., 2009). In turn, COX2-dependent release of MMP-9, vascular endothelial growth factor (VEGF), and fibroblast growth factors (FGF) support tumor vascularization and progression (Tjiu et al., 2009). In addition to macrophages, in BCC, there are increased numbers of tumor-infiltrating lymphocytes, 45% consisting of T-regs in peritumoral areas. Similarly, T-reg attracting chemokines such as CCL17, -18, -22, and CXCL12 are enhanced in BCC intra- and peritumorally (Omland, 2017). Thus, although BCC is an immunogenic tumor and well responsive to immunotherapeutic drugs, the infiltration of T-regs may generate an immunosuppressive niche hindering the response to checkpoint inhibitors (Omland, 2017).

### Merkel Cell Carcinoma

Merkel cell carcinoma (MCC) is sporadic but a highly aggressive skin cancer, and more than 80% of MCC are linked to Merkel cell polyomavirus (MCPyV) (Schadendorf et al., 2017). Intensive research has been done in MCC tumor biology, from discovering MCPyV in 2008 to the relevance of the UV-signature in MCPyV-negative MCC (Schadendorf et al., 2017). Further immunological studies finally led to the approval of checkpoint inhibitors for the treatment of MCC patients (Bradford et al., 2020). However, less is known about the role of the extracellular matrix in the aggressiveness of MCC. In primary MCC, an altered scaffold of collagen fibers is in the peritumoral area and possibly contributes to the invasiveness of this tumor. However, surprisingly, collagen expression was independent of prognosis (Laurito et al., 2021). Besides collagen, TNC expression is enhanced in grown tumors and localized at the MCC’s invasive front (Koljonen et al., 2005). Apart from those ECM components, MMPs are increasingly expressed in MCC. Increased expression of MMP-1 and MMP-3 are adverse prognostic factors (Massi et al., 2003), and MMP-10 and MMP-26 may associate with aggressive disease (Suomela et al., 2009). Furthermore, expression of MMP7, MMP10/2, and TIMP3 is associated with metastatic tumor spread (Fernández-Figueras et al., 2007). However, the functional significance of metalloproteinases in ECM remodeling in MCC is little explored. It was demonstrated that the skin cells most permissive for MCPyV infection are fibroblasts rather than Merkel cells, where this event may rather be rare. Most interesting, matrix remodeling by MMPs might support MCPyV infection of human dermal fibroblasts, although mechanistically is not clear how this occurs (Liu et al., 2016). Thus, infection of Merkel cells can be a secondary random event. In line with role of MMPs in supporting viral oncogenesis, expression of the Merkel cell polyomavirus large T antigen (LT) from MCC tumors induces MMPs expression in cells (Richards et al., 2015). Also, ADAM 10 and 17 are upregulated in MCPyV-positive primary MCC tumors where they disrupt cellular contacts leading to cell dissociation and motility underlying invasion (Nwogu et al., 2018).

In MCC, there is also an immune component. In MCC, overexpression of Chemokine (C-C motif) ligand 1 (CCL17/TARC) and C-C chemokine receptor type 4 (CCR4) leads to the attraction of many CD4^+^ regulatory T cells, Th2 and Th17 cells (Rasheed et al., 2018). Increased T-regs and M2 macrophages generate an immunosuppressive niche facilitating tumor progression and metastasis in MCC (Gaiser et al., 2018). However, a stronger infiltrate of CD8^+^ T cells in MCC is a favorable prognosticator (Touzé et al., 2011).

## ECM and Remodeling in Therapeutic Resistance

**FIGURE 2:**
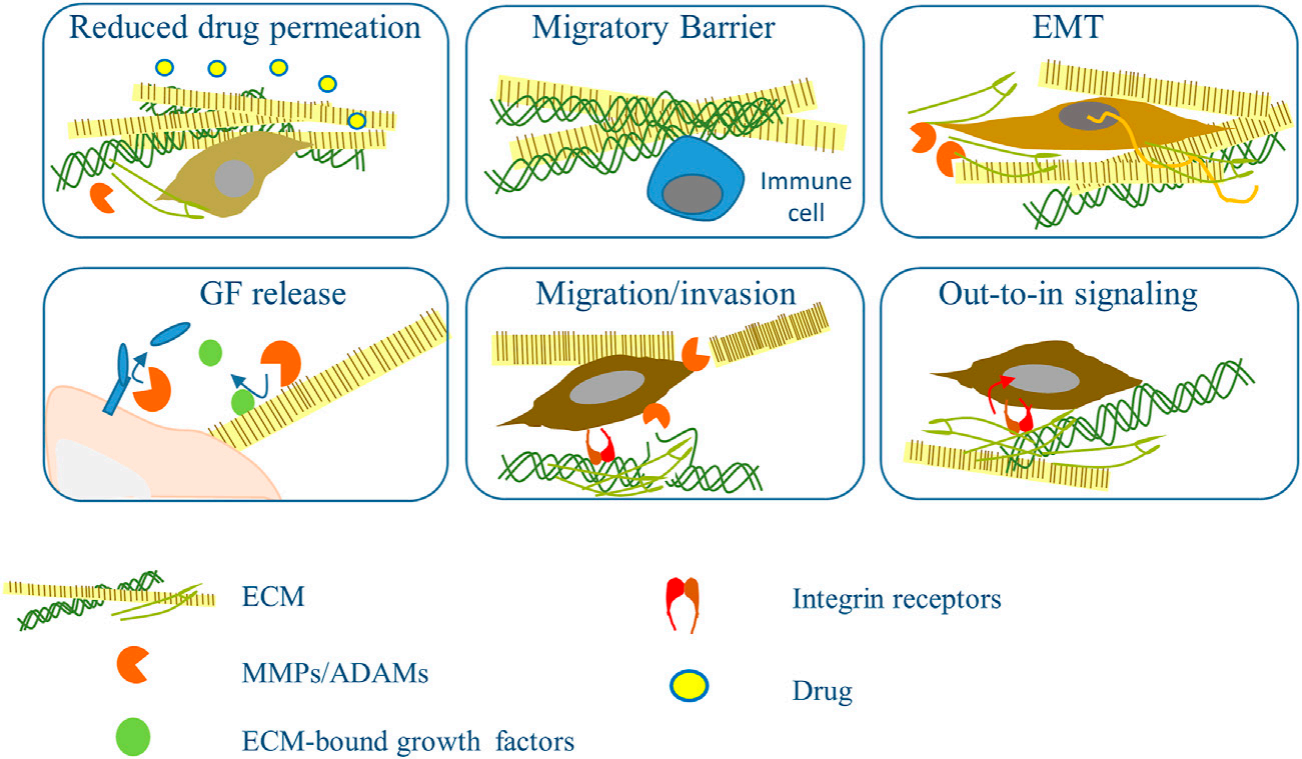
Mechanisms based on ECM remodeling and metalloprotease that influence response to therapy. Increased accumulation of ECM and its cross-linkage impedes access of the drugs to the tumor cells, the supply with nutrients and interferes with the migration of inflammatory cells. Dense ECM induces EMT that in turns fuels ECM accumulation. Active metalloproteinases (MMPs/ADAMs) from tumor and stromal cells help transformed cells cross ECM barriers and release cellular and ECM-bound growth factors and cytokines that play an essential role in chemotherapy resistance. Tumor cell contact with ECM mediated by integrins lead to the activation of an out-in pathway that increases survival signaling and helps circumvent the drug’s effect.

Although several new groundbreaking therapies have revolutionized the treatment of several cancers, including melanoma, the success is limited by developing various resistance mechanisms. ECM and its remodeling may account for the factors influencing efficacy and resistance to available anti-neoplastic drugs, radiation treatment, and some immunotherapies (Huang et al., 2021). Here we will point at some of the known data on the involvement of ECM and enzymes in resistance to targeted drugs used to treat skin cancers. Some of those mechanisms are summarized in Figure 2.

### MAPK Inhibitors

BRAF and MEK inhibitors have revolutionized melanoma therapy throughout recent years and are prescribed for patients with melanoma that harbor a BRAF mutation, present in about 60% of melanoma. By inhibition of the MAPK pathway, patients achieve previously unconceivable survival rates of about 76% (Davies et al., 2002; Dummer et al., 2018). However, after an excellent initial response, patients develop resistance over time and during treatment. Resistance is attributed to acquired new genetic mutations such as NRAS and MEK or overexpression of CRAF (Patel et al., 2021). Various other resistance mechanisms have been described, bypassing mutant B-RAF and reactivating PI3K/AKT and ERK signaling (Patel et al., 2021). These include epigenetic and transcriptomic mechanisms, for example upregulation of transcription factors, alterations in the tumor microenvironment, and immune, for example enhanced PD-L1 expression (Patel et al., 2021). A further contributor is collagen. In the initial phases of MAPK inhibitor treatment, increased amounts of collagen type I are deposited in the microenvironment to form a scaffold for the cells (Diazzi et al., 2020). Intravital imaging has localized MEK-inhibitor-resistant melanoma cells close to bundled collagen where they survive drug treatment; thus, matrix stiffness and reorganization are suggested to generate ECM niches fostering drug resistance (Brighton et al., 2018).

Moreover, although most ECM components are produced by stromal fibroblasts and CAFs, MAPK inhibitors can induce production of ECM molecules in melanoma cells, as detected for collagen and FN (Jenkins et al., 2015; Brighton et al., 2018; Diazzi et al., 2020; Girard et al., 2020). Melanoma cells with a loss of PTEN increased FN expression, thereby modifying signaling and attenuating the response to BRAF inhibitors (Fedorenko et al., 2016). Enhanced FN expression is only detected in BRAF inhibitor-resistant PTEN-null melanoma samples (Fedorenko et al., 2016). This is possibly due to the induced phenotypic shift in melanoma cells toward a mesenchymal phenotype. That, in turn, activates alpha5beta1 integrin/PI3K/AKT signaling, limiting the drug’s cytotoxic effects; this also results in enhanced expression of myeloid cell leukemia 1 (MCL1) protein, a mediator of evasion to targeted therapy (Fedorenko et al., 2015). By intravital imaging, Hirata et al. (2015) show that treatment of MAPK inhibitors activates peritumoral fibroblasts and induces enhanced FN whose binding to the integrin beta1/focal adhesion kinase/Src signaling provides an escape route for melanoma cells from treatment (Fedorenko et al., 2015; Hirata et al., 2015). Moreover, BRAF inhibitors activate ERK signaling in CAFs, leading to activation of beta-catenin and the secretion of POSTN. This protein, in turn, reactivates ERK signaling in melanoma cells under BRAFi pressure, further promoting resistance (Liu et al., 2022).

BRAF inhibitor-resistant cells display increased expression of active MMP-2, pointing at an increased matrix remodeling and consequently a higher invasive capacity (Sandri et al., 2016).

The capacity to cleave the extracellular matrix components using MMP-14 was associated with a resistant cell phenotype (Marusak et al., 2020). MMP-14 is upregulated in BRAF inhibitor-resistant cell lines and human tumor samples, and its upregulation is dependent on TGF-beta. Consequently, inhibition of MMP-14 or TGF-beta combined with a BRAF inhibitor reconstituted therapeutic efficacy (Marusak et al., 2020). In addition to matrix remodeling, MAPK inhibitor treatment led to rewiring of the cytoskeleton by enhancing actin fiber formation; thus, inhibiting ROCK1 (a modulator of the actin cytoskeleton) could represent a possible means to tackle resistance development (Smit et al., 2014).

Based on those mentioned earlier and several other data, combination therapy of a BRAF and PI3K inhibitor has been suggested (Fedorenko et al., 2016). Indeed, therapeutic efficiency could be reconstituted by combining a BRAF and FAK inhibitor (Hirata et al., 2015), although, upon both treatments, single or double therapy, cSCC developed, but with lower incidence in combination therapy (Flaherty et al., 2012). The development of these secondary tumors was the consequence of the paradoxical activation of the MAPK signaling (Gibney et al., 2013). The phenotypic shift induced in melanoma cells by these double treatments is dependent on YAP1 (yes-associated protein 1)- and MRTFA (Myocardin Related Transcription Factor A)-activity in resistant melanoma cells. Resistant cells are thus enabled to produce drug-resistant ECM with enhanced stiffness that counteracts treatment (Girard et al., 2020). In line with these, combinatorial treatment with a BRAF and a YAP1 inhibitor has been shown to decrease tumor growth (Girard et al., 2020). Enhancing ECM density and mechanical properties may compromise therapeutic efficacy by phenotypically shifting plastic melanoma cells, reduce tumor cell migration/invasion as less permissive (Wolf et al., 2013), by altering immune cells infiltration (see in the following paragraph), and by inducing myofibroblastic-CAF activation. A general explanation of the processes leading to ECM stiffening, its broad consequences on chemoresistance, and the clinical approaches to target it has recently been reviewed (Darvishi et al., 2022).

Enhancement of BRAF and MEK inhibitors can influence melanoma cells’ signaling and activate CAFs. By secreting hepatocyte growth factor (HGF), CAFs manage to reactivate MAPK and PI3K/AKT signaling contributing to the acquisition of resistance (Straussman et al., 2012; Wilson et al., 2012). Patients with HGF expression in the stroma show weaker therapeutic responses than patients without HGF expression (Straussman et al., 2012). Also, the secreted frizzled-related protein 2 (sFRP2), a Wnt antagonist secreted by peritumoral fibroblasts, weakens the response toward MAPK inhibitors (Kaur et al., 2016).

MAPK inhibitors induce an inflammatory reaction enhancing IL-1beta secretion by tumor-associated macrophages (TAMs) and production of CXCR-2 ligands by fibroblasts. Consequently, *in vivo* blocking IL-1R or CXCR2 signaling enhanced response to MAPK inhibitors (Young et al., 2017). TAMs affect melanoma cells by releasing TNF alpha, thereby sustaining survival signaling pathways in melanoma cells by enhancing MITF expression (Smith et al., 2014). Increased MITF expression enhances the secretion of ET-1, a reactivator of the ERK pathway, thus bypassing mutant BRAF and maintaining MAPK signaling (Smith et al., 2017). Moreover, in BRAFi-resistant invasive melanoma cells with MITF^low^, the expression of pentraxin 3 (an acute phase inflammatory glycoprotein) is high. It induces, likely via the TLR4, activation of the NFκB signaling pathway leading to enhanced invasion and expression of TWIST1 (Twist Family BHLH Transcription Factor 1) (Rathore et al., 2019). Melanoma-acquired resistance to targeted therapy displays a cross-resistance to checkpoint inhibitors (Haas et al., 2021). This cross-resistance has been suggested to be derived from a lack of CD103 + dendritic cells, whereas replenishing CD103 + dendritic cells eliminates cross-resistance. Based on their work, the authors propose a therapeutic protocol starting with checkpoint inhibitors followed by BRAF-inhibitor in BRAF mutated melanoma patients (Haas et al., 2021).

### Checkpoint Inhibitors

Tumor cells and the peritumoral stroma crosstalk generate a microenvironment that, from suppressive, becomes tumor promoting (Bhattacharjee et al., 2019). Due to a high burden of mutations caused by UV radiation and tumor-associated antigens, skin cancers show high immunogenicity, making them susceptible to checkpoint inhibitor therapy (Hodis et al., 2012; Pickering et al., 2014; Goh et al., 2016; Paulson et al., 2019). The tumor mutational burden has been suggested as a predictive marker for response to checkpoint blockade (Yarchoan et al., 2017). However, checkpoint inhibitors have demonstrated high response rates in various solid tumors: lung cancer, renal cell carcinoma, melanoma, SCC, BCC, and MCC (Brahmer et al., 2012; Paulson et al., 2019). In addition, melanomas display an enhanced expression of tumor-associated antigens predisposing melanoma cells for T-cell killing (Ilyas and Yang, 2015). Checkpoint inhibitors have revolutionized therapy for patients with metastatic melanoma and have demonstrated a curative potential (Robert et al., 2018). Compared to a 3 year overall survival rate of 12% with dacarbazine, the combined checkpoint blockade with ipilimumab (CTLA-4, cytotoxic T-lymphocyte-associated protein 4, inhibitor) and nivolumab (PD-L1, Programmed cell death-ligand 1, inhibitor) reaches about 60% (Robert et al., 2011; Wolchok et al., 2017). In skin cancer and other solid tumors, the infiltrate of T cells provides a predictor for a checkpoint inhibitor response, whereas infiltrating Tregs weaken therapeutic efficiency (Cristescu et al., 2018; Kugel et al., 2018). To circumvent the systemic side effects of those therapies, anti-PD-L1 and CTLA4 antibodies were conjugated to the extracellular matrix protein-PIGF-2 (placenta growth factor 2) to provide a local treatment in animal models of melanoma; this resulted in enhanced efficacy with reduction of tumor growth and improved survival (Ishihara et al., 2017).

Additionally, the conjugation with PIGF-2 led to a stronger infiltrate of CD8^+^ and CD4^+^ T cells, ensuring higher efficiency of the checkpoint inhibitor therapy (Ishihara et al., 2017). Similarly, the combination of checkpoint inhibitors and IL-2 with the collagen-binding domain of Willebrand factor A3 led to a mainly intratumoral accumulation of the drug. Thus, it lowered systemic side effects, reduced tumor growth, and enhanced T-cell infiltrate in a murine B16F10 melanoma model (Ishihara et al., 2019). In addition to limiting the systemic side effects, preventing the acquisition of resistance to checkpoint inhibitors remains the major challenge.

Although there is broader knowledge about the influence of the extracellular matrix on the development of resistance to BRAF/MEK targeted therapy, less is known on the impact of the ECM and its remodeling to checkpoint inhibitor resistance or its predictive potential for immunotherapy. However, recent studies showed that a marker for collagen type III fibrogenesis (PRO-C3) with high PRO-C3 in patients’ serum is a predictor of poor overall survival in metastatic melanoma upon treatment with anti-CTLA-4 and anti-PD-1 therapy (Jensen et al., 2018; Hurkmans et al., 2020). ECM proteins may regulate immune cells in the tumor microenvironment, and the ECM in the skin dramatically changes with age displaying reduced collagen deposition and enhanced fragmentation by the activity of MMPs (Russell-Goldman and Murphy, 2020). Indeed, patients under the age of 50 have a lower ratio of CD8^+^Tregs and are less responsive to anti-PD-1 than older patients, possibly due to the reduced ability of PMN-Myeloid-derived suppressor cells to migrate through the less dense and lax matrix to the tumor site (Kugel et al., 2018). That is in contrast with the observed inhibitory function of tumor-fibrosis on T cell infiltration and migration (reviewed in (Jiang et al., 2017). In cholangiocarcinoma, breast and pancreatic carcinomas preclinical tumor models, reverting stiffening of the dense peritumoral matrix using inhibitors of cross-linking enzymes improves anti-PD-1 therapy by enhancing the T cells infiltration and activation (Nicolas-Boluda et al., 2021). In contrast, desmoplastic melanoma, displaying a dense collagenous stroma, has a much higher response rate to anti-PD-1 therapy than other types of melanomas that is attributed to the presence of pre-existing T cell infiltrates in the invasive edge of the lesions (Eroglu et al., 2018). Altogether these data highlight the complexity of the tumor microenvironment and the induced cellular reactions that depend on the tumor type, context, and most likely on the temporal frame of therapy. Thus, a generalization concerning the role of peritumoral stiffening on tumor growth and therapeutic response cannot be made. Still, if a prediction of therapy efficacy should be made, it should be based on biophysical properties of the surrounding tissue that include not only stiffness, but also confinement, and the type of tumor and time of development.

Tumor evasion from immune control can be obtained by downregulation of major histocompatibility complex class-I and -II (MHC-I and MHC-II), which is detected in advanced but not early melanomas, and correlate to metastatic progression (Degenhardt et al., 2010). Among the factors regulating the expression of MHC class I complex is transforming growth factor-beta 1 (TGF-beta 1) thus being considered among the drivers of resistance (Lee et al., 2020). Considering the potent immunosuppressive effects the PD-L1/PD-1 pathway and of TGF-beta 1, a clinical trial designed to target both in solid tumors is ongoing and the results are expected soon (Merck/EMD Serono, NCT02517398). Interestingly, release by metalloproteinase shedding of the immunoreceptor ligand major histocompatibility complex class I chain-related molecule A (MICA) enables melanoma cells to escape immune surveillance (Nausch and Cerwenka, 2008). Consequently, inhibition of shedding that enhances MICAs expression restored NK–cell-mediated immunity against melanoma metastases in an *in vivo* tumor model (Ferrari de Andrade et al., 2020). In line with these data, MHC-II positivity on tumor cells is associated with response, progression-free and overall survival to anti-PD-1 therapy (Johnson et al., 2016). Melanoma susceptibility to immunotherapies is reduced by CAFs production of IL6 and induction of IL10 in melanoma cells, the latter inhibiting antigen-presenting cells and weakening the immune response (Terai et al., 2012; Simiczyjew et al., 2020). In further support of a resistance-promoting effect of CAFs, CAFs can mediate resistance to checkpoint blockade by secreting MMP-9, which can cleave PD-L1 from melanoma cells (Zhao et al., 2018). Interestingly, co-treatment of an MMP-2/-9 inhibitor and PD-1 or CTLA-4 blockade enhanced the therapeutic efficacy in the treatment of mouse models of melanoma and lung cancer (Ye et al., 2020).

The immune system has a pivotal role in developing cSCC, as shown by the frequent growth of cSCC in the skin of immunocompromised patients (Omland et al., 2018). In recent years, checkpoint inhibitors, particularly the PD-1 inhibitors Cemiplimab and Pembrolizumab, have been approved to treat unresectable or metastatic cutaneous squamous cell carcinoma (Shalhout et al., 2021). Human cSCC have enhanced expression of PD-1 and CTLA-4 (Gambichler et al., 2017), and blocking these proteins results in improved infiltration of T cells (CD4^+^ and CD8^+^), hindering the development of SCC in animal models (Belai et al., 2014). The response rates to PD-1 inhibitors in SCC patients are promising, but about 50% of patients do not benefit from checkpoint blockade or acquire resistance (Migden et al., 2018). Indeed, it has been reported that in SCC, PD-1-inhibition facilitates immune escape by inducing infiltration of T-regs (Dodagatta-Marri et al., 2019). In HNSCC, immune evasion upon PD-1-inhibition was promoted by the upregulation of Tim-3 (T cell immunoglobulin and mucin-domain containing-3 receptor; regulator of Th1 immunity) (Shayan et al., 2017).

Checkpoint inhibitors have also demonstrated high and long-lasting efficacy in MCC with a lately reported 42-months overall survival rate of 31% under avelumab. Most long-term survivors had MCC with a positive PD-L1 expression status (D'Angelo et al., 2020). The amount of MCPyV specific T cells increases with MCC tumor load displaying an enhanced expression of PD-1 and Tim-3 exhaustion markers (Afanasiev et al., 2013). Also, MCPyV-negative tumors showed elevated T cells and PD-L1 levels, indicating checkpoint inhibitors’ therapeutic efficacy in MCPyV-negative tumors (Afanasiev et al., 2013; Schadendorf et al., 2017). The most recent FDA approval is for checkpoint inhibitors in BCC treatment as second-line after hedgehog inhibitor therapy. Cemiplimab, a PD-1 antibody, treatment of BCC patients led to an objective response in 31% of patients, of which 6% showed a complete and 25% partial response (Stratigos et al., 2021). Since the history of checkpoint inhibitor therapy in non-melanoma skin cancer is very short, data on the acquisition of resistance and research about the underlying mechanisms are up to now very scarce. Thus, with the establishment of checkpoint inhibitors in non-melanoma skin cancer, subsequent long-term detailed studies are needed to analyze resistance drivers from the microenvironment in these tumor entities to obtain more durable responses.

## New Therapeutic Approaches

Treatment with BRAF and MEK and checkpoint inhibitors represent very efficient therapeutic options for patients with metastatic melanoma. However, the acquisition of resistance constitutes the principal and still unsolved clinical challenge. The reasons for the development of resistance are, as described beforehand, intensely investigated, and most approaches to tackle the evolution of resistance are based on a combinatorial approach, combining the established therapies with further complementing drugs.

Although ECM is suggested to contribute to the acquisition of resistance, very few therapeutic approaches focus on inhibiting resistance mechanisms deriving from the extracellular matrix. The critical, although the sometimes controversial, role of collagen deposition is described in the context of resistance to MAPK inhibitors. Collagen synthesis is strongly dependent on TGF-beta 1 (Liu et al., 2012a). In various solid tumors, including melanoma, inhibiting TGF-beta 1 reduced collagen synthesis *in vivo* (Diop-Frimpong et al., 2011). Moreover, Losartan, an antihypertension drug, has been shown to inhibit collagen synthesis and is generally well tolerated (Lim et al., 2001). As suggested in an early study, Losartan or neutralizing TGF-beta 1 has been suggested for the treatment of RDEB patients to prevent cSCC onset (Mittapalli et al., 2016). The related drug candesartan has been demonstrated to inhibit melanoma growth and vascularization in an animal model (Fujita et al., 2002). Thus, collagen inhibitors could represent an add-on therapy to BRAF and MEK inhibitors to impede or at least limit the development of resistance. Besides collagen, other therapeutic approaches may target FN, particularly the EDA and EDB domains whose appearance and upregulation in tumors impact tumor vascularization (Rybak et al., 2007). A monoclonal antibody against BC-1 (recognizing the EDB domain) fused with murine IL-12 could inhibit tumor progression in xenograft models of skin cancer. When applied to a small cohort of patients, about half achieved stable disease (Lo et al., 2007; Rudman et al., 2011). Another fusion antibody, L19-IL-2, targeting the EDB domain of FN combined with the IL2, was effective in an animal model and achieved stable condition in a clinical trial including patients with melanoma and renal cell carcinoma (Carnemolla et al., 2002; Eigentler et al., 2011). There have been a variety of early phase clinical trials with MMP inhibitors (Rudek et al., 2001; Winer et al., 2018), tumor-targeting immunocytokines (Johannsen et al., 2010), and fibronectin-targeting agents (Eigentler et al., 2011; Rudman et al., 2011; Danielli et al., 2015), in skin cancers which are comprehensively summarized in Table 1. However, up to now, especially for MMP inhibitors, dose-limiting toxicity is an unsolved clinical problem. The field is still open for more extended investigations to efficiently exploit the tumor microenvironment’s therapeutic potential and the ECM.

**TABLE 1:** Clinical trials with ECM-targeting agents.

Clinical trial	Molecular profile	Tumor type	Results	Toxicity	References
Oral COL-3 (NCT00001683)	Modified tetracycline derivative, MMP inhibitor	Melanoma, lymphoma, renal cell carcinoma	Disease stabilization in patients with a non-epithelial type of malignancy	Dose-limiting phototoxicity	Rudek et al. (2001)
Batimastat	MMP inhibitor	Malignant pleural effusion (melanoma, NSCLC, etc.), malignant ascites	Terminated in phase III due to local toxicity	Intolerable local toxicity	Reviewed by Winer et al. (2018)
131I-labeled Tenatumomab (NCT02602067)	Tenascin-C moAb labeled with iodine I 131	Skin cancer and others	Terminated, negligible uptake of the drug in tumors	n.a	n.a
AS1409 (NCT00625768)	BC1, Ab to FN ED-B linked to IL 12	Melanoma, renal cell carcinoma	Stable disease in 46% of patients; partial response in melanoma	adverse events grade 2 and 3	Rudman et al. (2011)
L19-IL2 (NCT01253096)	L19, Ab to FN EDB combined with IL 2	Metastatic melanoma, renal cell carcinoma and others	Stable disease; tolerable toxicity in renal cell carcinoma and other solid tumors	Tolerable toxicity	Johannsen et al. (2010)
L19-IL-2 + dacarbazine (NCT01055522)	L19, Ab to FN EDB combined with IL 2 and dacarbazine	Metastatic melanoma, renal cell carcinoma	28% of patients with objective response, including a complete response	Tolerable and reversible toxicity	Eigentler et al. (2011)
L19IL2+L19TNF (NCT02076633)	L19, Ab to FN EDB combined with IL 2 or TNF	Melanoma stage III or stage IVM1a	Reduced local tumor burden; prevented progression	Limited	Danielli et al. (2015)
L19IL2+L19TNF (NCT04362722)	L19, Ab to FN EDB combined with IL 2 or TNF	BCC or cSCC	Awaiting	n.a	n.a

Ab, antibody; moAb, monoclonal antibody; n.a., not available.
